# Obstetric neonatal emergency simulation workshops in remote and regional South India: a qualitative evaluation

**DOI:** 10.1186/s41077-021-00187-9

**Published:** 2021-10-14

**Authors:** Bella Zhong, Mahbub Sarkar, Nandakumar Menon, Shylaja Devi, Jayaram K. Budanoor, Naresh Beerappa, Atul Malhotra, Arunaz Kumar

**Affiliations:** 1 Monash University, Melbourne, Australia; 2 Monash Centre for Scholarship in Health Education (MCSHE), Faculty of Medicine, Nursing and Health Sciences, Monash University, Melbourne, Australia; 3 Ashwini Hospital, Gudalur, Tamil Nadu India; 4 Mandya Institute of Medical Sciences, Mandya, Karnataka India; 5 Tata Global Beverages Ltd. High Range Hospital, Munnar, Kerala India; 6 Department of Paediatrics, Monash University, 246 Clayton Road, Clayton, Melbourne, Victoria 3168 Australia; 7 Department of Obstetrics and Gynaecology, Monash University, Melbourne, Australia

**Keywords:** Asphyxia, Education, Emergency, Interprofessional, Low- and middle-income country, Maternity, Neonatal, Post-partum haemorrhage

## Abstract

**Background:**

Healthcare facilities in remote locations with poor access to a referral centre have a high likelihood of health workers needing to manage emergencies with limited support. Obstetric and neonatal clinical training opportunities to manage childbirth emergencies are scant in these locations, especially in low- and middle-income countries.

**Objectives:**

This study aimed to explore the factors, which influenced healthcare worker experience of attending birth emergencies in remote and regional areas of South India, and the perceived impact of attending the Obstetric and Neonatal Emergency Simulation (ONE-Sim) workshop on these factors.

**Design:**

Qualitative descriptive study using pre- and post-workshop qualitative surveys.

**Settings:**

Primary healthcare facilities in remote/regional settings in three states of South India.

**Participants:**

A total of 125 healthcare workers attended the workshops, with 85 participants completing the pre- and post-workshop surveys included in this study. Participants consisted of medical and nursing staff and other health professionals involved in care at childbirth.

**Methods:**

ONE-Sim workshops (with a learner-centred approach) were conducted across three different locations for interprofessional teams caring for birthing women and their newborns, using simulation equipment and immersive scenarios. Thematic analysis was employed to the free-text responses obtained from the surveys consisting of open-ended questions.

**Results:**

Participants identified their relationship with the patient, the support provided by other health professionals, identifying their gaps in knowledge and experience, and the scarcity of resources as factors that influenced their experience of birth emergencies. Following the workshops, participant learning centred on improving team and personal performance and approaching future emergencies with greater confidence.

**Conclusions:**

Challenges experienced by healthcare workers across sites in remote and regional South India were generally around patient experience, senior health professional support and resources. The technical and interpersonal skills introduced through the ONE-Sim workshop may help to address some of these factors in practice.

## Background

Maternal and neonatal mortality continues to substantial burden low- and middle-income countries (LMICs). Ninety-four percent of maternal deaths worldwide occur in low-resource settings, and most are due to preventable causes such as post-partum haemorrhage, infection and pre-eclampsia [1, 2]. In these settings, common causes of neonatal deaths include birth asphyxia, complications of premature birth and neonatal sepsis [3]. Despite a decline in recent years, maternal and neonatal mortality rates in LMICs like India remain high compared to those of high-income countries [2, 4].

Various systemic, healthcare worker and patient factors impact the delivery of quality intrapartum care in LMICs. These include issues with health infrastructure, availability of skilled workers, poor teamwork and patient communication [5]. Despite general increases in skilled birth attendant coverage in LMICs, low rates of skilled birth attendance and institutional delivery remain in rural areas [6]. Inadequate training has been identified as another significant barrier to quality midwifery care in LMICs [5]. Particular gaps include appropriate training for the management and referral of obstetric emergencies, especially for those working in remote areas without medical support [7].

In India, public institutions range from specialised urban hospitals to rural primary health centres (PHCs). Many PHCs lack basic infrastructure such as beds, toilets, drinking water, clean labour rooms and regular electricity [8]. Referral to higher levels of care is challenging in many poorly resourced rural and remote areas of India [7]. In this setting, the lack of training provided to healthcare workers is a significant factor influencing mortality rates [5].

Simulation-based education has emerged as an effective and popular training method for healthcare students and workers, especially in high-income countries [9]. Training can take place ‘in situ’ in the clinical setting or removed from the clinical setting in an off-site facility [10]. Major factors motivating the use of simulation-based education include focusing on patient safety, multi-professional teamwork and training for emergencies [11–13]. Simulation can develop a degree of clinical competence in the absence of real patient exposure and improve communication and teamwork skills to minimise errors that contribute to patient harm [9, 14]. In addition, simulation-based education can also facilitate deliberate practice of technical, problem-solving and decision-making skills [15–17]. Honing these abilities is particularly relevant to managing emergencies, which may be rare in clinical practice but can be practised in a safe environment through simulation [11].

There is growing evidence that simulation-based education results in improved knowledge, attitudes and performance of healthcare workers and improved patient outcomes [18–21]. Furthermore, medical simulation training appears to have a dose-response relationship with improved participant outcomes, in that more practice yields better results [22]. In the field of childbirth training, simulation has demonstrated improvements in both maternal and neonatal outcomes, with a reduction in complications [23–25]. However, these training facilities are not easily accessible to primary-secondary healthcare institutions in low- and middle-income countries. The current study addresses this gap through the Obstetric and Neonatal Emergency Simulation (ONE-Sim) workshops that can be implemented in various birth settings in LMICs [26], including those in rural areas.

The ONE-Sim workshops use low technology, low-maintenance mobile simulation equipment that can be transferred to distant sites and quickly set up to implement training for multi-professional teams (more details of the workshop in the methods section). Participants are afforded hands-on experiences of managing birth emergencies, both maternal and foetal/neonatal using simulators, with a focus on scenarios relevant to their setting, to improve communication and teamwork skills in addition to technical and problem-solving proficiency [26]. We previously demonstrated the application of the ONE-Sim workshop in secondary level districts and metropolitan hospitals. Through this study, we introduced ONE-Sim workshops to institutions in rural and remote South India, and aimed to further explore the factors that influenced participants’ past experiences of birth emergencies in these scantily resourced settings. Studying these factors was thought to provide an insight into how to address the challenges in clinical practice. Based on previous similar research [26], there was a gap in the management of clinical emergencies in a team-based environment. Hence, we attempted to address some of those gaps in learning through the ONE-Sim workshop. We also studied the impact of the workshop on these factors as perceived by the participants. The research questions were as follows:

1. What are the factors affecting successful management of obstetric and neonatal emergencies in remote to regional areas of South India?
2. How does the ONE-Sim workshop contribute to managing obstetric and neonatal emergencies in their practice, as perceived by participants?

## Methods

### Study design

The study follows a qualitative descriptive design with a view that this design can present a comprehensive descriptive summary of participants’ experiences and perspectives, without abstract rendering of data [27]. Participants completed a pre-workshop qualitative survey that explored their views on the factors that affected how they managed obstetric and neonatal emergencies. Drawing on previous research in another setting, where patients identified a few challenges related to the lack of availability of medical staff and need for structured training [26], the hands-on ONE-Sim workshop addressed these concerns. Given the difference in context (i.e. remote settings), we perceived that it was important to explore the challenges participants in remote settings encountered prior to attending the ONE–Sim workshop. Following the ONE-Sim workshop, they completed another qualitative survey that unpacked their perspectives on how the workshop contributed to managing obstetric and neonatal emergencies.

### Study settings

ONE-Sim workshops were conducted at three geographically distant, independently functioning public healthcare providers across three states in remote to regional areas of South India: (i) a district training facility in Mandya, Karnataka state; (ii) a rural hospital in Gudalur, Tamil Nadu state; and (iii) a regional district hospital in Munnar, Kerala state. The common factor for all these sites was the remoteness of location and poor access to a referral centre at least 100 km away from the nearest tertiary referral centre. The sites catered to rural population. The cultural backgrounds and spoken language used for day-to-day interactions with colleagues and clients were different for the three sites. Each healthcare site conducted between 300 and 1000 births per year, whilst all three had fewer than 20 permanent/rotating healthcare workers through the centres, with casual staff at some sites. All sites provided primary care but also had facilities to conduct caesarean sections. After hours, an obstetrician and paediatrician were available to be on call; however, only one senior doctor was employed for each of these specialities.

### Participant characteristics

Workshop participants comprised of local healthcare workers involved in childbirth (both permanent and casual staff) and medical and nursing students on their birth unit rotations at the time. In Indian healthcare education, rather than having a separate midwifery course, nurses usually undergo specialised midwifery training in the fourth and final year of their course. The doctors who participated were consultants and junior healthcare staff from obstetrics and paediatric teams, and a family physician.

### Recruitment

Participants were sent explanatory statements a few weeks prior to attendance of the workshop to acquaint them with the details of the study. On the day of the workshop, participants were verbally briefed by the administrative team organising the workshop about the study aims and methods, including data collection, analysis and reporting. They had the choice to attend the workshop without participating in the study by not completing the surveys. In this sense, participation in the study was voluntary.

### ONE-Sim program

The ONE-Sim program was a 4-h training workshop, as described above, conducted at each site by the lead facilitators in conjunction with local medical facilitators. The design and content of workshops were developed based on an iterative convergent design, using feedback regarding each site’s available facilities, the scope of practice and local protocols to direct clinical management.

Design of the ONE-Sim workshop was informed by situated learning theory [28], which views learning as situated in its context, activity and culture. Learning may be embedded in everyday activity and is mostly unintentional rather than deliberate. It is a process that is both constructional and transformational in nature, which may occur from clinical and life experiences or through a focused simulated learning session. It is dynamic, influenced by culture, through the usual social relationships and interactions with colleagues, peers and clients. The learning process depends on guidance, support, co-construction and re-conceptualisation of practice. Aligning with this theory, we considered contextual factors (e.g. challenges faced by the participants in their clinical practice) and included hands-on activities in our workshop. The activities were contextualised considering participants’ cultural settings. We engaged local faculties in the design and delivery of the workshop to connect the learners using context that applies to their practice.

The ONE-Sim workshop is an adaptable, culturally responsive learning package. Participants may vary in their individual social and cultural backgrounds or organisational work culture influencing their challenges in clinical practice. It may also have an impact on how they learn and apply that learning in their own context. The fluidity and adaptability of the workshop are maintained through flexible scenario construct, culturally acceptable interactions such as the use of local language, engaging local faculties and finding pragmatic team-based solutions to suit the setting. The workshop content prompted them in finding common solutions as a team, sharing best clinical practices, and, eventually, driving change. Our workshop introduces an opportunity for learners and faculties to ‘step back’ and review their problems when facing an emergency, reflect upon their management of obstetric and neonatal complications, and engage further in advancing knowledge and skills, jointly, as a team.

The ONE-Sim program was led by an obstetrician (AK) and a neonatologist (AM), with support from local faculties that included obstetricians, paediatricians and nursing educators employed by the relevant healthcare institution. AK and AM spent some time prior to the workshop to understand the work-based arrangements, facilities for birth attendants and referral process involved for each site. They interacted with medical-nursing staff regarding the roles of birth attendants and arrangements available in case of an emergency. This background work assisted in developing rapport with the local faculties who co-designed the scenarios and co-facilitated the teaching and debrief. The objective of co-facilitation was to engage learners better by providing direct translation of the teaching in the local language, and to develop a home-based interprofessional medical-nursing faculty for conducting future in-house training workshops.

A Prompt Flex simulator (Limbs and Things, Bristol, UK) and a neonatal resuscitation model, Newborn Anne (Laerdal Medical, Stavanger, Norway), were used as simulation equipment. These were packed together in a suitcase and were easily portable from site to site, requiring 15-30 min to set up prior to the workshop.

Following demonstration of birth on the simulators and familiarisation with the equipment, participants underwent independent skills training with feedback provided at each skill station. Stations consisted of conducting normal labour, recognising and managing obstructed labour, breech birth, shoulder dystocia and postpartum haemorrhage and resuscitation of an asphyxiated newborn. Participants then practised management of obstetric and neonatal emergencies on the simulators in teams during facilitated scenarios. These included a variety of team-based clinical situations, as well as some where conflict within the teams was anticipated. This prompted discussions about management and the divisions of responsibility and helped encourage a team-based learning approach. Finally, participants contributed to a clinical discussion and debrief, emphasising key learning messages from the workshop.

### Data collection

Data were collected using paper-based pre- and post- workshop qualitative surveys. The surveys were developed by the researchers and facilitators (AK and AM) of the workshop. The pre-workshop survey started with a range of demographic questions (e.g. profession, years of clinical experience, exposure to simulation component in their education). The survey then included an open-ended question which asked them to describe challenging experiences they encountered during obstetric or neonatal emergencies and how they addressed the challenges. The qualitative data gathered with this question identified factors (e.g. barriers and facilitators) affecting successful management of obstetric and neonatal emergencies in the remote to regional areas of South India (RQ1). The post-workshop survey concerned the participants’ learning in relation to knowledge, attitudes and skills, as well as their perceptions of how the workshop contributed to managing emergencies in their own practice (RQ2). This survey consisted of three open-ended questions that asked participants to reflect on their experiences of participating in the workshop. They were asked about how the workshop would impact their handling of emergency scenarios and their clinical practice, in addition to the advantages and disadvantages of participating as a team.

The surveys were distributed and collected by the administrative staff organising the workshop. These were completed on-site. Participants could respond in either English or their local language. Responses provided in local languages were translated verbatim. All written responses were converted into electronic documents and used for analysis.

### Data analysis and reflexivity

Data were analysed using the thematic analysis approach [29]. The process started with reading data multiple times to ensure familiarity and develop a deeper understanding of the data. Initially, the authors, BZ and AK, independently coded the data and generated themes inductively. BZ and AK then met to discuss their analyses and the different insights they brought to their interpretations of the data. Based on their discussion, they negotiated meanings and interpretations and came up with refined themes and sub-themes. These were reviewed by AM and MS and further discussed as a team to compare, contrast and negotiate team-based interpretations of the themes and sub-themes generated. The discussions included a notion of reflexivity where the researchers examined their positioning within the research. AK and AM are experienced in conducting simulation workshops and qualitative research, with clinical backgrounds in obstetrics and neonatology. They facilitated the workshops, but had no prior relationship with any participants, and participant knowledge of the researchers concerned their credentials only. BZ is an entry-level qualitative researcher, and MS is an experienced qualitative researcher and health professional educator. BZ and MS were not involved in the design and conduct of the workshop. Bringing these different perspectives to data analysis supported the rigour of the study.

## Results

### Professional characteristics

A total of 125 participants attended four workshops conducted over 4 days in October 2019. Eighty-five participants completed both the surveys voluntarily (participation rate 68%), consisting of 59 nurses, 7 doctors, 15 students and 4 other health professionals involved during childbirth. The years of clinical experience of participants ranged from 1 to 31 years, with a median of 6 years. None of the participants had attended a simulation session in their health professional course prior to the workshop.

### Factors influencing participants’ experience of emergency situations

In the pre-workshop survey, participants described a range of factors that influenced their experience of managing obstetric and neonatal emergency situations. Four major themes were generated: (a) supporting the patient, (b) recognising the support of healthcare team, (c) limited expertise and experience and (d) limited resources.

#### Support for the patient

Supporting the birthing women that included relationship and rapport building was described by participants as challenging in birth emergencies. In particular, students and junior staff considered managing the woman’s concerns and providing reassurance to be a major aspect of their role.

I was with the mother, who had just birthed, and my objective was to reassure her as best as I could. … I continually assured the mother that her baby was receiving the most appropriate care at the time, and I think this provided some level of relief for the mother.

However, the connection participants formed with women in their care could produce emotional strain, especially when participants struggled with empathy for the woman’s pain or lingering guilt over complications, such as when one nurse wrote:

Even after the episiotomy 3kg baby was delivered with lots of difficulty. Baby then had one hand weakness. I was tensed. Even now I feel sad and worried.

Participants also described how patients’ limited knowledge which led to lack of co-operation challenged them to provide support. For example,

One day a girl 15 years old came with labour pain … She had lack of knowledge regarding delivery pain and what she [had] to do. … She doesn’t know how to push and didn’t have much prior information and knowledge regarding labour … we staff all together tried to support her but she was not co-operative.

#### Recognising the support of healthcare team

Participants identified beneficial aspects of teamwork in the management of emergencies, with division of labour enabling an efficient and coordinated response, and the expertise of other team members being reassuring. Medical support was requested upon the identification of a concerning situation, or the failure of nursing/midwifery management. In challenging situations, the descriptions of many nurses were similar to: ‘I didn’t know what to do… Asked for help. Informed doctors’.

Referrals were central to handling emergencies, with women and newborns commonly moved to referral centres for further management. For many participants, their most challenging experiences concerned these conditions that could not be managed with the skills and resources that were available to them. One midwife gave the example:

While feeding the baby with [expressed breast milk], baby dropped the heart rate and colour also changed. I didn’t know what to do. I straightway started the CPR and gave oxygen … After review by the doctors baby diagnosed with blockage of [oesophagus] needing surgery. Baby was then referred to referral centre for surgery.

There were also instances where team factors either failed to mitigate or otherwise exacerbated the stress of a situation, due to junior staff and students perceiving a lack of support from senior clinicians, lack of leadership causing disorganisation and panic, as well as when medical staff were delayed in arriving at the scene. As one student stated:

It was frustrating and stressful thinking that there was something wrong, but not being able to do anything about it as the doctor did not share my concern. … the staff left the room without doing any observations/monitoring. I found this very stressful…

#### Limited expertise and experience

Participants commented on their own lack of expertise in managing neonatal and obstetric emergencies. According to them, incomplete knowledge and skills, combined with inexperience handling such situations, were liable to result in unsuccessful management or complications. A nurse gave an example: ‘I had not dealt with shoulder dystocia earlier, and delivered the baby with brachial plexus palsy’.

Participants described how their inexperience with emergency situations and limited knowledge of management procedures made them stressed. For example, a student commented:

I was present for a shoulder dystocia delivery during my 3rd year labour and delivery rotation. It seemed very chaotic at the time but that was because I didn't know the steps of dealing with it yet. I think the most stressful part was not knowing what to do to help effectively.

Participants experienced added pressure to perform the appropriate management in unfamiliar emergency situations, which produced fear and panic. In the words of a junior doctor:

I was new to [the] clinical world … I felt like what is happening, if patient collapses on table [then] what will I do. These kinds of questions were screaming inside my skull.

#### Limited resources

Inadequate resources were a major factor impacting participants’ ability to provide adequate care was. This primarily included a shortage of personnel numbers to care for both the woman and baby or to cope with the number of births and a specific lack of medical staff. Nurses emphasised the latter at the primary centres, who recalled being unable to obtain medical support during births:

… paediatrician is necessary but in PHC level, night time no doctors, no paediatrician [is available].

Other issues cited were a lack of medications and consumables, and occasionally basic infrastructure, such as when one nurse described:

… some time no medication, no electrical [power], not proper working radiant warmer.

### Impact of the ONE-Sim program in management of emergencies

Participants generally commented positively about their experience of the ONE-Sim program, with many expressing the wish to attend further revision sessions in the future. Three major themes were generated from their responses, concerning participants’ learning and perceptions of the workshop in relation to the management of obstetric and neonatal emergencies in their practice: (a) improving team performance, (b) improving individual performance and (c) approaching emergency situations with confidence.

#### Improving team performance

Following the workshop, participants shared a resolve to strengthen their teamwork. They reflected on the importance of recognising when to call for help and considered ways to assist and teach their colleagues. Enhancing teamwork through simulation was described as a way to improve team performance. As one nursing student noted:

… these scenarios are complex and high risk and require team management in real life and should therefore be practised in teams.

The exercise helped define each role’s responsibilities in these situations and raise the capabilities of team members to similar levels.

It is helpful learning as a group so that everyone is on the same page when there is a real emergency ... everyone has the same level of knowledge.

These components were thought to improve the cohesiveness of the team and optimise their ability to collaborate. In the words of a nurse:

As a team we now know we can work together and the ways which work best for us.

#### Improving individual performance

Improvement of individual performance was highlighted through gaining knowledge and practical experience, and learning in a group environment. Many participants remarked that the workshop content was novel to them and believed that their increased knowledge would help them recognise emergency situations and understand the underlying condition and necessary management. One student reflected on her learning with the words:

The workshop was structured in a way that clearly outlined each step to take in a number of emergency situations making it easier to remember appropriate steps under the stress of an emergency. This clear and structured approach will help me respond in clinical practice.

Participants appreciated the practical aspect of the workshop, with advantages being the opportunity to practise clinical emergency skills and manage challenging scenarios in a safe environment. The application of theoretical learning through simulation was seen as a useful adjunct that increased understanding and retention of information. As a nurse stated:

Actually when studying and doing my theory block I don’t understand. But today by doing practise I learned, it is fixed in my mind.

Participation in the workshop as a group garnered largely positive feedback, as individuals felt supported to ask questions and approach scenarios with the pooled knowledge and experience of the team. Additional benefits included being able to consolidate learning through repetition and observe others. One student commented:

Working in a team is good for confidence. People support each other by offering suggestions. Then watching people solve the issue/problem solve is great for learning. See different positives and negatives of the action decided.

However, the potential for individual experiences to be hindered by the group-learning format was also recognised. For example, participants reported that the time available to individually engage with the simulation was reduced, the delivery of information was affected by noise, and as one student wrote:

Difficult to see demonstrations at times – difficult to tailor more/less training to individuals who may need it.

#### Approaching emergency situations with confidence

Participants expressed increased confidence in their abilities to manage emergencies after the workshop. Whereas emotions of fear, discomfort and doubt were predominant in the pre-workshop surveys, post-workshop responses demonstrated that participants believed they were better prepared to respond to difficult situations. In addition, there was newfound confidence to face emergencies and perform steps to manage the emergency. In the words of a nurse:

I was really scared about newborn resuscitation, but now am really happy after attending this, I have a confidence now that I can do newborn care and also in emergency PPH and shoulder dystocia, and also the steps of shoulder dystocia.

Some respondents also referred to their clinical confidence in independent management where team support was unobtainable. Nurses felt better equipped to provide appropriate treatment in the event that doctors were unavailable or delayed in arrival.

PPH management very useful. Learnt how to give fluids and manage situations without doctors.

## Discussion

**Fig. 1:**
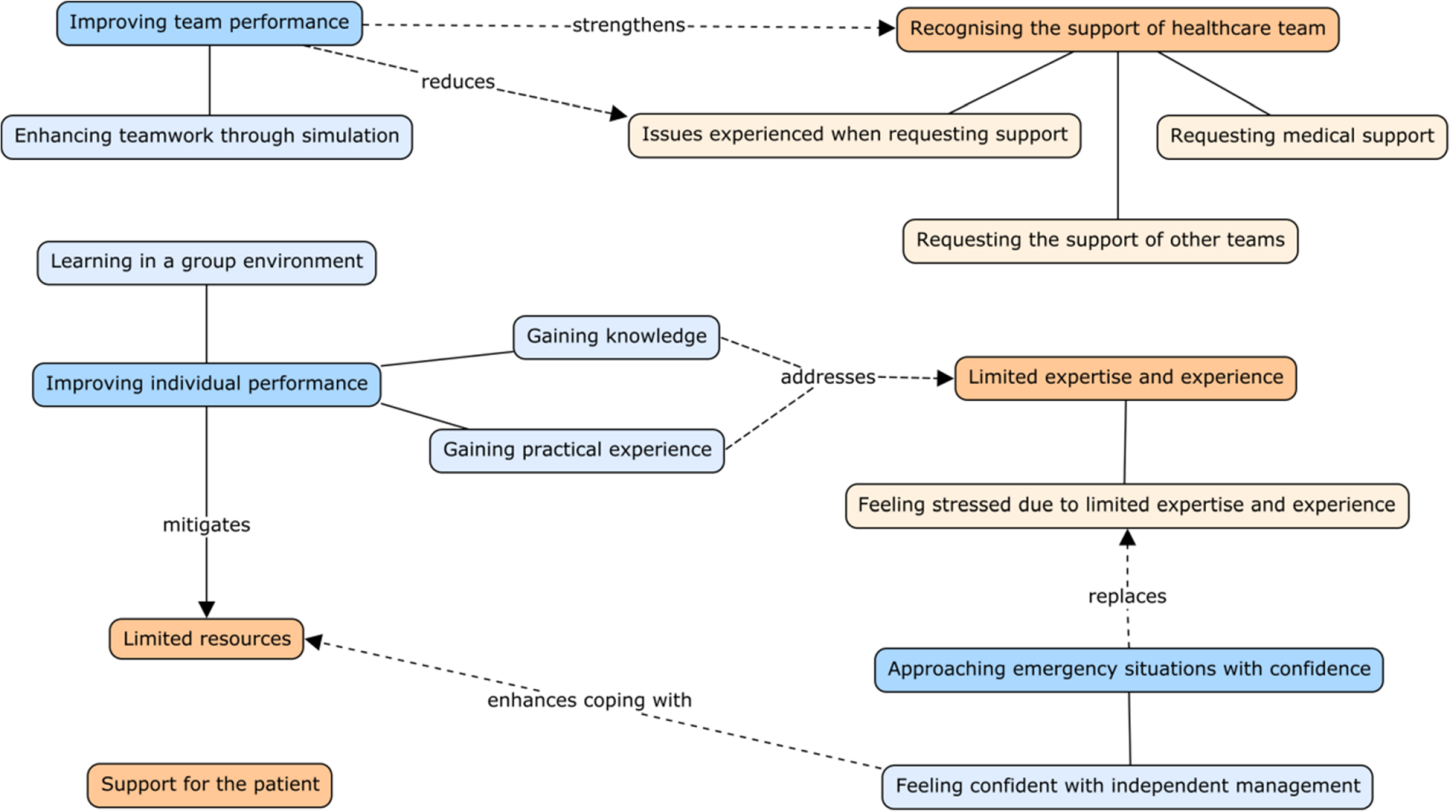
The contribution of learning from the ONE-Sim workshop (blue) to the factors influencing management of obstetric and neonatal emergencies (orange)

In the context of high neonatal and maternal mortality rates in India, the ONE-Sim workshops aimed to address the need for further nursing and medical education, especially in the rural setting. Simulation workshops were conducted across three remote or regional primary health centres in South India. The study aimed to assess the factors, which influenced participants’ past experiences of obstetric and neonatal emergencies, and the potential impact of the workshop on these factors, as perceived by participants. This was addressed through thematic analysis of data from pre- and post-workshop surveys. Healthcare workers identified their support of the patient, support provided by the healthcare team, their limited expertise and experience, and the limited resources as being prominent factors that influenced their experience of birth emergencies. Participant feedback following the workshop centred on improving team and individual performance, and approaching future emergencies with greater confidence. Figure 1 illustrates the relevance of workshop learning to managing challenges in practice. Based on participants’ perceptions, the ONE-Sim workshop has the potential to address factors, which may impede or promote successful management of birth emergencies.

Participant reflections on their workshop learning also demonstrated the concepts of situated learning, which underpinned the ONE-Sim design [28]. They valued learning through simulations based on the context and culture of their clinical practice to improve individual and team performance. In particular, participants appreciated the hands-on opportunities to apply their knowledge and skills in problem-solving scenarios. The group participation emulated the relationships and interactions between the healthcare team when managing real-birth emergencies. Participant responses highlighted the sharing of knowledge and experience amongst the group to reach common solutions and learn through observation and collaboration with others. This model of simulated learning was perceived to be appropriate and effective for learners, in the context of birth attendance in rural and remote India.

The results of this study add to a relatively small but growing body of literature regarding the use of simulation-based education in low- and middle-income countries. Previous studies have explored the use of simulation training involving the care of the woman, neonate, or both in birth emergencies, across South Asia and Sub-Saharan Africa [26, 30–36]. These studies have assessed simulation-based education workshops across all four Kirkpatrick levels, a model for evaluating training programmes [37]. In addition to measuring participants’ reactions, learning and behaviour, there has been increasing focus on the translation of training to patient outcomes. However, many of these interventions are based in suburban and metropolitan hospitals, with very few studies involving rural and remote settings, where the provision of emergency obstetric and neonatal care may be more challenging. This paper offers a perspective on conducting simulation-based training in these peripheral locations.

As reported previously [26], ONE-Sim is a mobile workshop that can be delivered across various locations and settings and may be suitable for birth attendants in more remote locations. The current study expands on the relevance of the ONE-Sim workshop in rural to remote areas. Participants were predominantly midwives, who comprise the vast majority of personnel in rural PHCs. Consistent with existing studies, their responses highlight the challenges posed by the lack of staff, training and resources in these settings [5, 8]. However, participants also perceived their learning from the ONE-Sim workshop to contribute to their team and personal performance in practice, equipping them with greater confidence to manage emergencies in this (often challenging) environment. This paper, therefore, builds on previous knowledge of the ONE-Sim workshop, demonstrating its ability to be refocused to specific rural and remote settings, and the relevance of workshop learning to managing the particular challenges of attending births in this context.

Prior to the workshop, teamwork was mainly regarded as a positive element of managing emergencies. Requesting support from medical staff or other teams, division of labour and the expertise of other team members were found to alleviate the stress of the situation. The learning outcomes of the simulation encouraged participants to further build upon the strengths of teamwork. In the post-workshop survey, participants recognised the importance of calling for help and understanding the responsibilities of their role in an emergency. The issues identified when requesting support may also be mitigated by the workshop experience. Participating senior staff anticipated using new strategies to coordinate the team and guide junior staff during emergency situations.

As one of the best practices for simulation-based education, team training can develop leadership and communication skills necessary for effective multidisciplinary teams [12, 16, 21]. Additionally, simulation training promotes a sense of group identity and trust, which were reflected in participants’ feedback of improved team cohesion [16]. These attitudes and behaviours can reduce the panic and disorganisation in the absence of leadership and the lack of support junior staff sometimes felt from senior clinicians. By simulating team management and conflict scenarios, the workshop was able to teach behavioural skills, which enhance team performance.

Other major contributors to stress were recognised by healthcare workers to be their own lack of expertise, in addition to scarcities of staffing and equipment. In the pre-workshop survey, many participants related lack of knowledge, skills and experience with neonatal and obstetric emergencies to unsuccessful management or complications. Students struggled with being unfamiliar with emergencies and their management protocol, whilst healthcare workers expressed fear and panic under the pressure of managing these situations. These factors may be positively influenced by the workshop, as participants perceived their knowledge of managing obstetric and neonatal emergencies to have increased following attendance. Participants commented on the application of simulation and participation as a group in consolidating and retaining theoretical learning. These results are consistent with the existing body of literature that simulation-based education is effective in improving the knowledge of healthcare workers and the transfer of theory to the clinical context [17–19, 21]. Navigating through challenging scenarios in a safe supportive environment through a step-by-step approach, at repeated intervals, will likely help reduce future stress associated with birth emergencies [38].

Improving the personal performance of healthcare workers may alleviate the burden of a lack of staffing resources. Participants hoped to improve on past practice through greater preparation and efficiency during emergencies along with gaining knowledge and technical skills. In the absence of systemic change, enhancing individual capabilities may help healthcare workers manage future situations where they may encounter a shortage of personnel. In addition, where management of emergencies without medical support was unavoidable, nurses’ newfound confidence in their abilities can reduce the stress associated with providing independent treatment.

## Limitations

A limitation of this study is its inability to measure the clinical outcomes in participant practice following workshop participation. Whilst this would be beneficial for evaluating the effectiveness of simulation training, it was considered beyond the scope of this study, which focused on the self-perceived impact of workshop engagement and application to their experience of birth emergencies. Additionally, the use of surveys lacks depth in comparison to alternative research methods such as interviews. Some of the survey responses were of limited length, which increased the risk of over-interpretation by the researchers. However, this method was able to efficiently collect data from a large number of workshop participants across a variety of clinical backgrounds and settings. Further research is planned to continue to evaluate the long-term impact of such training, including how training attendance supports participants to address the contextual challenges they encounter. Other areas for further investigation include how the sustainability of these workshops can be maintained, and the barriers to incorporation of simulation-based education in clinical practice in these settings.

## Conclusions

A diverse range of factors influence healthcare workers’ experiences of obstetric and neonatal emergencies in remote and regional South India. Aspects of their relationship with patients, individual perception of lack of expertise, back-up support, and lack of resources were identified as barriers to successful management of such situations. The technical and interpersonal skills training introduced through the ONE-Sim workshop remain relevant to addressing these factors in practice across regional to remote settings. Regular workshops may be useful for skills development and maintenance in healthcare workers involved in births. Further evaluation of translation to practice and effect on patient outcomes would be warranted in assessing the effectiveness of simulation-based education for birth emergencies in low-resource settings.

## Data Availability

The datasets generated and analysed during the current study are available from the corresponding author on reasonable request.

## Abbreviations

ONE-Sim: Obstetric and Neonatal Emergency Simulation
LMIC: Low- and middle-income country
PHC: Primary health centre
PPH: Postpartum haemorrhage
CPR: Cardiopulmonary resuscitation
